# Immunogenicity and Cross Protection in Mice Afforded by Pandemic H1N1 Live Attenuated Influenza Vaccine Containing Wild-Type Nucleoprotein

**DOI:** 10.1155/2017/9359276

**Published:** 2017-01-22

**Authors:** Andrey Rekstin, Irina Isakova-Sivak, Galina Petukhova, Daniil Korenkov, Igor Losev, Tatiana Smolonogina, Tatiana Tretiak, Svetlana Donina, Svetlana Shcherbik, Tatiana Bousse, Larisa Rudenko

**Affiliations:** ^1^Institute of Experimental Medicine, Saint Petersburg, Russia; ^2^Centers for Disease Control and Prevention, Atlanta, GA, USA

## Abstract

Since conserved viral proteins of influenza virus, such as nucleoprotein (NP) and matrix 1 protein, are the main targets for virus-specific CD8+ cytotoxic T-lymphocytes (CTLs), we hypothesized that introduction of the NP gene of wild-type virus into the genome of vaccine reassortants could lead to better immunogenicity and afford better protection. This paper describes in vitro and in vivo preclinical studies of two new reassortants of pandemic H1N1 live attenuated influenza vaccine (LAIV) candidates. One had the hemagglutinin (HA) and neuraminidase (NA) genes from A/South Africa/3626/2013 H1N1 wild-type virus on the A/Leningrad/134/17/57 master donor virus backbone (6 : 2 formulation) while the second had the HA, NA, and NP genes of the wild-type virus on the same backbone (5 : 3 formulation). Although both LAIVs induced similar antibody immune responses, the 5 : 3 LAIV provoked greater production of virus-specific CTLs than the 6 : 2 variant. Furthermore, the 5 : 3 LAIV-induced CTLs had higher in vivo cytotoxic activity, compared to 6 : 2 LAIV. Finally, the 5 : 3 LAIV candidate afforded greater protection against infection and severe illness than the 6 : 2 LAIV. Inclusion in LAIV of the NP gene from wild-type influenza virus is a new approach to inducing cross-reactive cell-mediated immune responses and cross protection against pandemic influenza.

## 1. Introduction

Influenza A viruses are highly contagious respiratory pathogens that annually cause up to 250,000 fatal cases [1]. Vaccination remains the most effective tool for controlling influenza. Current seasonal influenza vaccines need to be reformulated and administered every year due to continuous antigenic drift of the influenza virus; therefore attempts are made to develop universal influenza vaccines capable of protection against broad range of influenza viruses of human or avian/animal origin, including those with pandemic potential [2]. However, it is unlikely that such universal influenza vaccine will be on a market within next decade, and in the meantime it would be wise to improve the existing influenza vaccines which already have cross-reactive potential. The emergence of a new pandemic H1N1 influenza virus in 2009 (A/California/2009) and the threat of transmission of avian viruses to humans have stimulated research and development of live attenuated influenza vaccines (LAIVs) against newly appearing influenza viruses [3]. The World Health Organization (WHO) recommends the development and stockpiling of influenza vaccines for all potential pandemic strains [4]. Formulations of LAIV against pandemic influenza strains, including H1N1, H5N1, H9N2, H2N2, H7N3, and H7N9, have recently been tested in preclinical and phase I clinical studies [5–7]. The majority of these LAIV strains had a 6 : 2 genome formulation; that is, their hemagglutinin (HA) and neuraminidase (NA) genome segments came from avian or human wild-type viruses with pandemic potential, while the other six internal genome segments were derived from the cold-adapted (ca) A/Leningrad/134/17/57(H2N2) master donor virus (MDV) (Len17). This genome composition has been historically used for generating vaccine reassortants based on cold-adapted viruses as a backbone [8, 9].

The conserved viral proteins of influenza virus, such as nucleoprotein (NP) and matrix 1 protein (M1), are the main targets recognized by host virus-specific CD8+ cytotoxic lymphocytes [10, 11]. We therefore hypothesized that inserting the NP gene of pandemic wild-type virus into the genome of vaccine reassortants, using reverse genetics techniques, could lead to greater immunogenicity and hence afford better protection [12].

This paper describes preclinical studies of two new pandemic H1N1 LAIV candidates obtained by reverse genetics. The first candidate had a 6 : 2 genome formulation, with HA and NA genes derived from pandemic A/South Africa/3626/2013 (H1N1) and other genes from Len17 MDV. The second candidate had a 5 : 3 formulation, with HA, NA, and NP genes from A/South Africa/3626/2013 (H1N1) and other gene segments from Len17 MDV.

The study compared the following: the biological properties of the two candidate strains (growth characteristics, ts and ca phenotype); their safety (replication efficiency in the upper and lower respiratory tract of mice); their immunogenicity (ability to induce antibodies and cell-mediated responses after one or two doses); and their protective efficacy (against homologous and drifted wild-type viruses).

## 2. Materials and Methods

### 2.1. Materials

#### 2.1.1. Viruses

The wild-type virus A/South Africa/3626/2013 (H1N1) (SA/wt) was obtained from The National Institute for Biological Standards and Control (NIBSC, UK) repository; A/New York/61/2015 (H1N1) (NY/wt) viruses were obtained from the repository of the Centers for Disease Control and Prevention (CDC) (Atlanta, GA, USA). H1N1 LAIV reassortant viruses, with a 6 : 2 or 5 : 3 genome composition, were generated by standard plasmid-based reverse genetics, as described elsewhere [13]. Both viruses inherited their HA and NA genes from SA/wt H1N1 virus and their PB2, PB1, PA, M, and NS genes from Len17 MDV. The gene encoding viral nucleoprotein was derived either from the MDV (for the 6 : 2 reassortant) or from the SA/wt virus (for the 5 : 3 reassortant). Both viruses were fully sequenced and were found to be identical except for the NP gene. The Len17 MDV was used as a control in virus replication kinetics and neuroinvasion studies in mice. A recombinant A/PR/8 7 : 1 rg (NP-SA) (H1N1) virus, containing the NP of SA/wt virus and the remaining 7 genes from the PR8 backbone, was generated by reverse genetics and used in challenge experiments. All viruses were propagated in 10-11-day-old clean chicken embryos supplied by «Sinyavino» poultry farm (Kirovsk Area, Leningrad Region, Russia). Eggs were incubated for 48 hours at 33°C and harvested viruses were stored in aliquots at −70°C. The median infectivity (50% egg infectious dose (EID_50_)) was determined according to the method of Reed and Muench [14] and expressed as the mean log_10_EID_50_/ml.

#### 2.1.2. Mice

Female C57BL6 mice of 6–8 weeks of age were purchased from the laboratory breeding nursery of the Russian Academy of Sciences “Stolbovaya” in Moscow Region.

### 2.2. Methods

#### 2.2.1. In Vitro Characterization Studies

Temperature-sensitive (ts) and cold-adapted (ca) phenotypes of the viruses were determined by titration in eggs at different temperatures: 38°C compared with 33°C for the ts phenotype and 26°C compared with 33°C for the ca phenotype. Eggs were inoculated with 10-fold virus dilutions and incubated for either 48 hours (at 33°C and 38°C) or 6 days (at 26°C). The growth characteristics of the H1N1 LAIV viruses were also analyzed in Madin-Darby canine kidney (MDCK) cells. For this, cell monolayers were infected in triplicate with the LAIV H1N1 6 : 2 and LAIV H1N1 5 : 3 viruses at a multiplicity of infection (MOI) of 0.01; 150 *μ*l of the media was collected every 12 hours and stored at −70°C prior to titration. Virus titers determined in eggs were expressed in log_10_EID_50_/ml and those in MDCK cells in terms of median tissue culture infective dose (log_10_TCID_50_/ml).

#### 2.2.2. Viral Replication Kinetics and Neuroinvasion in Mice

To determine virus infectivity and neuroinvasion, mice were lightly anesthetized with ether and inoculated intranasally with 50 *μ*l of phosphate-buffered saline (PBS) containing 10^6^ EID_50_ of either LAIV, MDV, or SA/wt, divided equally between the nostrils. Viral load was measured in respiratory and brain tissues collected on days 3 and 6 after infection. Tissue homogenates were prepared using a small bead mill TissueLyser LT (QIAGEN, Germany), in 1 ml of sterile PBS containing antibiotic-antimycotic (Invitrogen, UK); the clarified supernatants were titrated in eggs at a temperature that allowed determination of virus levels. In addition, the brain homogenates were tested for the presence of viral RNA by conventional RT-PCR assay using universal primer pairs that amplify short regions of viral genes, as described elsewhere [15]. The SA/wt virus was used as a positive control in this assay.

#### 2.2.3. Immunogenicity and Protection Studies in Mice

Groups of thirty animals were inoculated intranasally with 10^6^ EID_50_ of either LAIV H1N1 6 : 2 or LAIV H1N1 5 : 3 vaccine candidates, in a volume of 50 *μ*l. Control mice received 50 *μ*l of PBS intranasally. Blood samples and nasal washes were collected before immunization and 21 days after immunization. On day 21, the animals also received a second intranasal inoculation of the virus given on day 0. On day 42 after the primary immunization, a third blood sample and nasal wash were taken. Hemagglutination inhibition (HAI) antibody titers were determined in the individual serum samples collected on days 0, 21, and 42. Sera were tested against homologous SA/wt and heterologous NY/wt virus, using 0.75% chicken red blood cells. An enzyme-linked immunosorbent assay (ELISA) was used to detect IgG antibodies in serum specimens, as described previously [16]. The ELISA end-point titers were expressed as the highest dilution that gave an optical density (OD) greater than two times the mean OD plus standard deviation (SD) of negative control samples. The HAI and IgG titers were log_2_-transformed to conduct statistical analyses.

To assess protection and cross protection, all animals were infected intranasally on day 42 with approximately 10^6^ EID_50_ of SA/wt, NY/wt, or A/PR/8 7 : 1 rg (NP-SA) (H1N1). Four animals from each group were euthanized on day 45, and the respiratory and systemic organs were harvested for virus titration. Virus titers were expressed as the mean log_10_EID_50_/ml ± SD. The body weights of the remaining mice were measured daily up to 14 days after challenge.

#### 2.2.4. Virus-Specific CD4+ and CD8+ T-Cell Response

Spleens from mice immunized with either LAIV H1N1 6 : 2 or LAIV H1N1 5 : 3, and from mock-immunized mice, were collected on days 21 and 42 after inoculation. Single spleen cell suspensions were prepared and frozen in liquid nitrogen until the assay. Virus-specific CD4+ and CD8+ Т-lymphocytes were determined by flow cytometry. Virus-specific T-cells were identified by conventional intracellular cytokine (gamma interferon) staining. Briefly, the 1 × 10^6^ of splenocytes were stimulated in vitro with SA/wt virus at 1.5 MOI in RPMI-1640 medium not containing fetal bovine serum. After 1-hour stimulation, fetal bovine serum was added to give 10% final concentration and cells were further incubated overnight. As a negative control, spontaneous gamma interferon production was determined by adding the appropriate volume of RPMI-1640 nutrient medium to cells; the resulting measurement was subtracted from the data obtained for virus-stimulated cells. The following monoclonal antibodies were used for staining: (1) Live/Dead Fixable Far Red Dead Cell Stain Kit (Invitrogen); (2) PE-Cy5 Anti-Mouse CD8a (BD Pharmingen); (3) Rat Anti-Mouse CD4/L3T4a-FITC (Beckman Coulter); and (4) PE Rat Anti-Mouse Gamma Interferon (BD Pharmingen). CD4+IFN*γ*+ and CD8+IFN*γ*+ T-cell populations were analyzed within live cells gate.

#### 2.2.5. CTL In Vivo Assay

This assay was performed as described elsewhere [17], with minor modifications. Briefly, to prepare target cells, splenocytes from naive C57BL6 mice were harvested and red blood cells lysed. Half of the cells were loaded with influenza SA/wt virus at 0.5 MOI for one hour in complete RPMI-1640 medium without fetal bovine serum (FBS) at 37°C. The remaining cells were mock-loaded with diluted normal chicken egg allantoic fluid in culture medium. After incubation, mock- and virus-loaded cells were washed and stained with 20 mM and 2 mM of carboxyfluorescein succinimidyl ester (CFSE), respectively. Next, target cells were washed with Hanks balanced salt solution and filtered thought 40 um cell strainer (BD Bioscience, USA). Then mock- and virus-loaded target cells were mixed in a 1 : 1 ratio. 1.5 × 10^6^ target cells were administered in 100 *μ*l to anesthetized mice on day 42 after the primary immunization, by retroorbital injection. Next day, the mice were sacrificed and splenocytes were harvested and processed by flow cytometry. Cytotoxicity was presented as virus- to mock-loaded cell count ratio.

*Statistical analysis* of the results was carried out using GraphPad Prizm 6 and Statistica 10 software. The nonparametric Mann–Whitney Test and Kruskal-Wallis test were applied for data comparison. *p* value < 0.05 was considered to be statistically significant.

### 2.3. Ethics Statement

The animals and chicken embryos were handled in accordance with the* Manual for Laboratory Animals and Alternative Models in Biomedical Research* [18]. Mouse experiments were reviewed and approved by the Institutional Local Ethical Committee. Fertilized eggs used for virus propagation were discarded in an appropriate manner, according to Russian Sanitary-epidemiological rules SP 1.3.2322-08 (approved 28 Jan 2008).

## 3. Results and Discussion

### 3.1. In Vitro Characterization of LAIV H1N1 6 : 2 and 5 : 3 Reassortants

**Figure 1:**
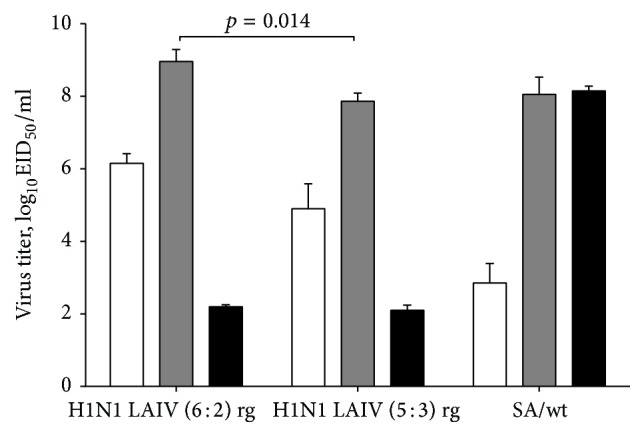
Infectious viral titers in eggs at different temperatures. Viruses stocks propagated in eggs at the permissive temperature (33°C) were titrated 3–5 times by end-point dilutions at the permissive or nonpermissive temperatures (26°C and 38°C). The bars represent arithmetic mean virus titers at indicated temperature ± standard deviations (SD) (T-lines). *p* value is shown for the two H1N1 LAIVs being compared at 33°C (Mann–Whitney *U* test). White bars: 26°C (for the ca phenotype); grey bars: 33°C (optimal temperature); black bars: 38°C (for the ts phenotype).

**Figure 2:**
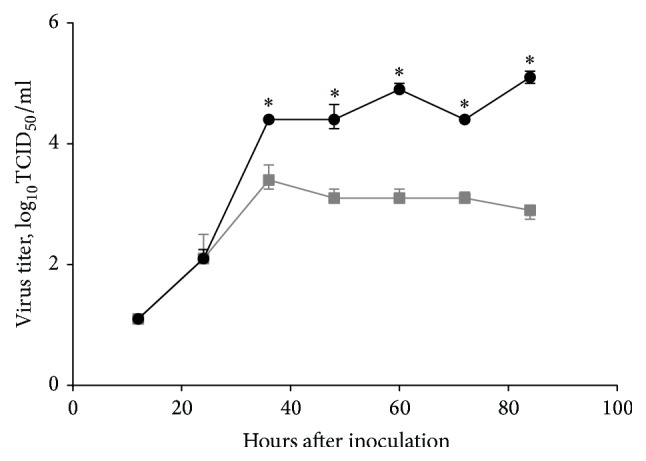
Kinetics of virus replication in MDCK cells (MOI 0.01). Cell monolayers were infected with studied viruses at a multiplicity of infection (MOI) of 0.01 in triplicate and incubated at the permissive temperature (33°C). Culture supernatants were collected every 12 hours and stored at −70°C prior to titration by 50% tissue culture infective dose (TCID_50_). Graphs show median titers with interquartile range (T-lines). Statistically significant differences between studied viruses are indicated with an asterisk (*p* < 0.05, Mann–Whitney *U* test). Black circles: H1N1 LAIV (6 : 2) rg; grey squares: H1N1 LAIV (5 : 3) rg.

The infectious titers of the 6 : 2 and 5 : 3 LAIV H1N1 reassortants in eggs indicated that both viruses possess the ts/ca phenotype: they grew poorly at 38°C (viral titer compared with 33°C > 5.0 log_10_ lower) and replicated efficiently at 26°C (viral titer compared with 33°C < 3.0 log_10_ lower). In contrast, the SA/wt parental virus grew poorly at 26°C, while its titer at 38°C was identical to that at 33°C (Figure 1). The titer of the LAIV H1N1 5 : 3 virus was significantly lower than that of the 6 : 2 reassortant at 33°C, suggesting that the NP gene of SA/wt virus could negatively affect viral growth. This effect was even more pronounced when the viruses were grown on MDCK cells. Although the cell monolayers were inoculated with an identical amount of each virus (MOI = 0.01), the 6 : 2 reassortant had significantly higher titers from 36 hours after inoculation (Figure 2).

### 3.2. Vaccine Replication Kinetics and Neuroinvasion in Mice

**Figure 3:**
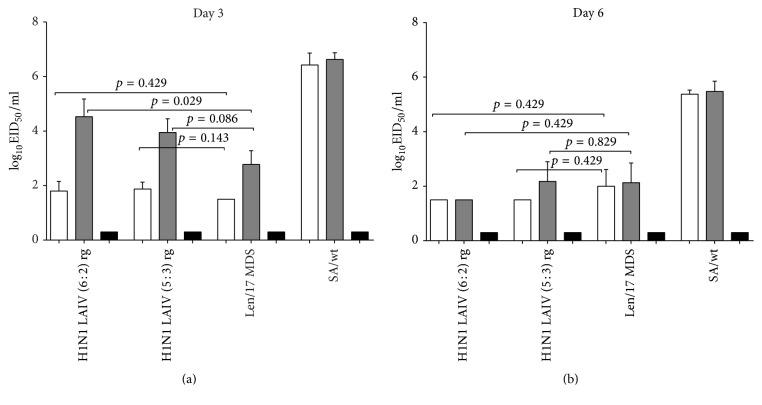
Replication of H1N1 LAIVs, MDV, and wild-type influenza viruses in upper and lower respiratory tract of mice and neuroinvasion. Groups of 8 mice were inoculated i.n. with 10^6^ EID_50_ of each virus; four mice from each group were euthanized on either day 3 or day 6 p.i. Mouse respiratory and brain tissues were collected and homogenized, and viral titers were determined by end-point titration in eggs. The virus titers are expressed as the mean log_10_EID_50_/ml ± SD (T-lines). The limit of virus detection was 1.2 log_10_EID_50_/ml, indicated by a dotted line. White bars: lungs; grey bars: nasal turbinates; black bars: brain. *p* values were calculated by Mann–Whitney *U* test.

The LAIV H1N1 6 : 2, LAIV H1N1 5 : 3, and Len17 MDV influenza viruses, administered at a dose of 6 log_10_EID_50_, replicated in mouse lung to a level of 1.8–2.2 log_10_EID_50_/ml on day 3 after inoculation (Figure 3(a)). On day 6, replication of the cold-adapted viruses in the lungs had decreased to 1.5–1.8 log_10_EID_50_/ml. The reproduction of the new reassortants and the MDV in the nasal turbinates showed the same trend – 2.8–4.2 log_10_EID_50_/ml on day 3 and 1.5–2.4 log_10_EID_50_/ml on day 6. In contrast, the wild-type virus, A/South Africa/3626/2013 (H1N1), showed up to 10^3^–10^4^ higher replication in the lungs and noses of mice on day 3 (6.9 log_10_EID_50_/ml), and on day 6 it was still replicating at high levels in the upper and lower respiratory tracts (5.8 log_10_EID_50_/ml) (Figure 3(b)). There was no significant difference between the LAIV viruses and the MDV with regard to replication in the lower respiratory tract of mice. Both the 6 : 2 and the 5 : 3 reassortants were indistinguishable from the MDV in terms of replication in the lungs and noses of mice on day 6 after inoculation. No infectious virus was found in the brain tissue of immunized mice in any of the studied groups on days 3 and 6 (in undiluted samples). To detect the presence of low amount of virus in the brain tissues we extracted RNA from brain homogenates followed by RT-PCR with universal primers targeted to conserved regions of influenza A viruses. The assay is very sensitive and can detect influenza A virus at a dose as low as 10^−1.68^ EID_50_ [15]. Brain tissues from all studied groups were RT-PCR negative, regardless of the primer pairs used in the reactions, whereas all reactions were positive for the control SA/wt virus (data not shown). Thus, both vaccine candidates were shown to be safe and identical to the Len17 MDV in terms of replication in upper and lower respiratory tract of mice; both lacked neuroinvasive capacity and failed to replicate in mouse brain.

### 3.3. Immunogenicity and Cross-Reactive Antibody Response

**Figure 4:**
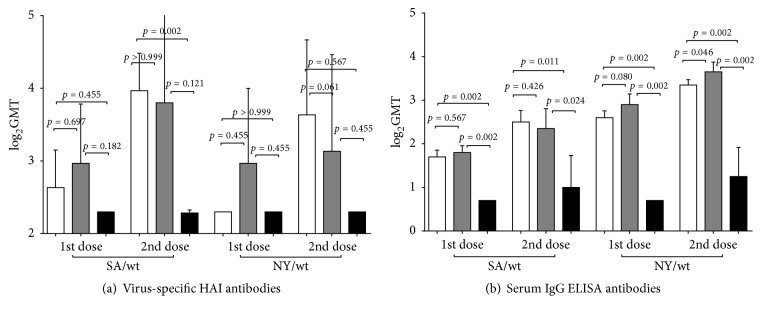
Antibody immune responses following immunization of mice with H1N1 LAIVs. Groups of 12 mice were inoculated i.n. with two doses of 10^6^ EID_50_ of each LAIV virus 21 days apart or mock-vaccinated. Mice sera were collected from 6 mice in each group 21 days after the first dose and 21 days after the second dose. HI (a) and IgG ELISA (b) tests were performed using egg-grown whole viruses SA/wt and NY/wt as antigens. Bars represent geometric mean with SD calculated from log_2_-transformed HI and ELISA titers. Statistical significance of differences between the vaccine groups was estimated by the Mann–Whitney test. White bars: H1N1 LAIV (6 : 2) rg; grey bars: H1N1 LAIV (5 : 3) rg; black bars: Mock.

Both LAIV candidates were found to be immunogenic in mice inoculated with 10^6^ log_10_ EID_50_ (Figure 4). Virus-specific HAI antibodies to homologous SA/wt virus were detected in both vaccine groups after first vaccination, but levels were not statistically significant between 6 : 2 and 5 : 3 reassortants. As expected, after the second dose, the levels of HAI antibodies to homologous virus substantially increased in the sera of mice that received either the 6 : 2 or the 5 : 3 reassortant; however, only in the group given the 6 : 2 variant was the difference in level between first and second dose statistically significant. Both vaccines induced detectable levels of HAI antibodies to drifted NY/virus after the second dose; however, the data were statistically significant only for the 6 : 2 LAIV (Figure 4(a)).

In both vaccine groups, substantial levels of homologous and cross-reacting IgG antibodies were detected in sera after doses 1 and 2 (Figure 4(b)). The differences between experimental and control groups were statistically significant in all cases; the 6 : 2 and the 5 : 3 vaccines induced comparable levels of IgG antibodies to homologous SA/wt and heterologous NY/wt virus.

### 3.4. Cross Protection of H1N1 LAIVs

Mice immunized twice with the 6 : 2 or 5 : 3 LAIV were completely protected against death after all three challenge viruses (Table 1). In contrast, mock-immunized mice had mortality rates of 100%, 83,3%, and 33,3% after challenge with NY/wt, SA/wt, and NP-SA, respectively. As shown in Table 1, the challenge viruses actively replicated in the lungs of the mock-immunized animals (7.2 log_10_ EID_50_ for SA/wt; 5.8 log_10_ EID_50_ for NY/wt; 5.8 log_10_ EID_50_ for NP-SA).

Mice immunized with the 6 : 2 LAIV had a greater maximum weight loss (up to 18.3%) and a lower reduction in lung viral titers (0.8–2.4 log_10_EID_50_/ml) than mice immunized with the 5 : 3 LAIV (up to 3.3% weight loss and 1.9–3.0 log_10_EID_50_/ml reduction in lung virus titers). Thus, both variants provided protection against challenge with homologous and drifted pathogenic H1N1 viruses, but the cross protection against infection and severe illness afforded by the 5 : 3 vaccine candidate was superior.

### 3.5. Virus-Specific CD4+ and CD8+ T-Cell Response

**Figure 5:**
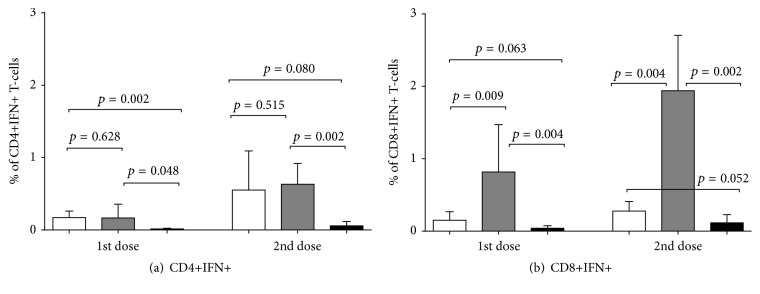
Virus-specific CD4+ and CD8+ T-cell responses following immunization of mice with H1N1 LAIVs. Groups of 12 mice were inoculated i.n. with two doses of 10^6^ EID_50_ of each LAIV virus 21 days apart or mock-vaccinated. Mice splenocytes were collected from 6 mice in each group 21 days after the first dose and 21 days after the second dose. Levels of IFN*γ*-secreting CD4+ T-cells (a) and CD8+ T-cells (b) were determined after stimulation with SA/wt whole-virus. Bars represent arithmetic mean with SD. Statistical significance of differences was estimated by the Mann–Whitney *U* test. White bars: H1N1 LAIV (6 : 2) rg; grey bars: H1N1 LAIV (5 : 3); black bars: Mock.

Significant increases in CD4+ T-cells were observed in both vaccine groups, in comparison with the control group, starting from the first dose (Figure 5(a), *p* = 0.002;  *p* = 0.048). After the second dose, the number of CD4+ cells was 9.5-fold higher for the group given the 6 : 2 LAIV and 10.9-fold higher for the group given the 5 : 3 LAIV, in comparison with mock-infected mice. No statistically significant difference in the virus-specific CD4+ T-cell response was observed between mice immunized with the 6 : 2 or the 5 : 3 variant after the first or second dose (*p* = 0.628;  *p* = 0.515, resp.). In contrast, CD8+ cell responses were much higher in mice given the 5 : 3 reassortant than in those that received the 6 : 2 variant, after both first and second doses (Figure 5(b), *p* = 0.009;  *p* = 0.004, resp.). Thus, including the wild-type NP gene in the reassortant increased production of specific CD8+ T-lymphocytes to wild-type influenza virus.

### 3.6. Antiviral Cytotoxicity In Vivo

**Figure 6:**
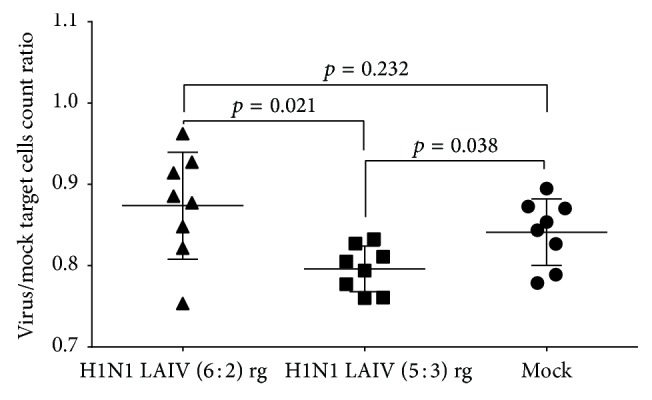
Cytotoxic activity of CTLs following immunization of mice with H1N1 LAIVs. Groups of 8 mice were inoculated i.n. with two doses of 10^6^ EID_50_ of each LAIV virus 21 days apart or mock-vaccinated. Splenocytes from naive C57BL/6 mice were loaded with SA/wt virus and adoptively transferred by retroorbital injection to vaccinated mice on day 21 after the second dose. The next day, specific cytotoxicity was measured and represented as the ratio of the count of virus-loaded target cells to that of control target cells. Bars represent arithmetic mean values with SD. Statistical significance of differences was estimated by Mann–Whitney *U* test. Triangles: H1N1 LAIV (6 : 2) rg; squares: H1N1 LAIV (5 : 3) rg; circles: Mocks.

To assess functional activity of the induced CLTs we measured the survival of target cells infected with wild-type virus relative to mock-infected controls, 16–18 hours after adaptive transfer into LAIV-immunized mice. The mean ratios of virus- to mock-loaded cell counts were 0.87 for mice immunized with the LAIV 6 : 2 reassortant, 0.79 for those given the 5 : 3 variant, and 0.84 for the nonimmunized group (Figure 6). In comparison with the naive group, more effective killing of wild-type infected cells was seen in mice immunized with the 5 : 3 LAIV, which included the NP gene segment from SA/wt virus.

### 3.7. Discussion

Safe, immunogenic, and protective prepandemic and pandemic influenza vaccines are urgently needed. Vaccination is primarily directed to inducing protective antibodies to the hemagglutinin and neuraminidase of the influenza virus. However, induction of a T-cell-mediated immune response should also be seriously considered. Cytotoxic T-lymphocytes (CTLs) are directed against more conserved influenza virus proteins, such as M1 and NP, which means that the CTL response is more cross-reactive. CTL responses do not prevent initial infection but, once primed, exert their effect on infected cells to aid recovery and restrict disease progression [19].

In recent years, several new approaches to developing such “universal” CTL-inducing vaccines have been tried. A recombinant M2 protein, with three tandem copies of M2e (3M2e), NP epitopes, and a hepatitis B virus core (HBc), has been studied as a virus-like particle (VLP) adjuvant to influenza vaccine in mice. It produced robust M2e-specific antibodies and cellular immune responses. Remarkably, 3M2e-NP-HBc VLP vaccine demonstrated effective cross protection against lethal challenge with pandemic 2009 H1N1 and HPAI H5N1 viruses [20].

In Balb/c mice, administration of a single dose of A/NP+M2-rAd adenovirus vector vaccine via the intranasal route was superior to intramuscular immunization in inducing mucosal responses and in protecting against challenge with highly virulent H1N1, H3N2, or H5N1 influenza virus. Intranasally vaccinated mice not only survived but had little morbidity and lower lung virus titers. Protection was observed as early as 2 weeks after immunization and lasted 10 months, as did antibodies and lung T-cells with activated phenotypes. Virus-specific IgA antibodies showed correlation with protection but were not essential, as demonstrated in studies with IgA-deficient animals. The authors suggested that such NP- and M2-expressing rAd vaccines could be used in the interval between emergence of a new virus strain and availability of a strain-matched vaccine [21].

In another study, DNA plasmids and recombinant vaccinia viruses expressing the conserved NP, polymerase basic 1 (PB1), and M1 proteins from influenza virus strain A/Beijing/30/95 (H3N2) were generated. BALB/c mice were immunized intramuscularly with a single vaccine based on NP, PB1, or M1 alone or with a combination vaccine based on all three antigens. They were then challenged with lethal doses of the heterologous influenza virus strain A/PR/8/34 (H1N1). Vaccines based on the three antigens provided complete or partial protection against challenge with 1.7 times the median lethal dose (LD_50_) of the PR8 strain. Of the three individual antigens, NP-based vaccines showed the greatest protective effect, inducing protection against 5 and 10 times the LD_50_. The authors concluded that universal influenza vaccines based on a combination of NP, PB1, and M1 induced a strong immune response and might be an alternative approach to addressing future influenza virus pandemics [22].

Live attenuated influenza vaccines are another attractive CTL-inducing option for control of pandemic influenza. They have a number of clear advantages: no needle injection is involved; they naturally stimulate both humoral and cell-mediated immunity; they prevent transmission of influenza viruses; they are of low cost; and they provide immediate protection to some extent [5, 23].

An interesting report by Chen et al. [24] evaluated the effect of seasonal H1N1 infection, seasonal trivalent inactivated vaccine (s-TIV), and seasonal trivalent live attenuated influenza vaccine (s-LAIV) administered before immunization with a pandemic LAIV in mice. Two doses of pandemic LAIV induced a cellular immune response and robust ELISA and neutralizing antibody responses that were associated with complete protection against challenge with pandemic H1N1 virus. A single dose of pandemic LAIV induced a cellular and ELISA response but not a neutralizing antibody response and gave incomplete protection against pandemic H1N1 virus challenge. Primary infection with seasonal H1N1 influenza virus followed by a dose of pandemic LAIV resulted in cross-reactive ELISA antibodies and a robust cellular immune response that was also associated with complete protection against pandemic H1N1 virus challenge. A lower-magnitude but similar response associated with partial protection was seen in mice that received a dose of seasonal LAIV followed by pandemic LAIV. Mice that received a dose of s-TIV followed by pandemic LAIV did not show any evidence of priming. The authors concluded that prior infection with a seasonal influenza virus or seasonal LAIV primed mice for a robust response to a single dose of pandemic LAIV, which was associated with protection equivalent to two doses of the matched pandemic vaccine [24].

A set of prepandemic and pandemic H1N1, H2N2, H5N2, H7N3, and H7N9 LAIVs were developed in our laboratory based on the fully attenuated, safe, cold-adapted master donor virus A/Leningrad/134/17/57 (Len/17); they were tested in preclinical and phase I clinical studies [3, 6, 25–31]. These reassortant LAIVs have 7 : 1 or 6 : 2 genome formulation, with surface glycoproteins HA, or HA and NA, derived from wild-type virus and the remaining genes from the MDV. The LAIVs were generated by classical reassortment, which is time-consuming, taking up to three months to obtain a vaccine candidate. The more modern reverse genetics (rg) technique allows more timely generation of LAIV reassortants with any genomic structure [32].

The Len/17 LAIV backbone originates from a virus isolated in 1957. Despite the greater antigenic conservancy of internal virus structures, a positive immune pressure on their immunodominant epitopes had been shown [33]. To overcome the problem of inducing CTLs targeted to the epitopes which no longer exist in circulating viruses, several approaches in LAIV design can be applied, such as generation of mosaic genes with updated epitopes compositions or whole gene renewal strategy. Nucleoprotein is the best target for such gene renewal strategy, since NP gene of Len/17 MDV does not control attenuated phenotype of the virus [13] and due to the presence of multiple immunodominant CTL epitopes within viral NP. Thus, in this study we generated and evaluated in a mouse model a pair of H1N1 LAIVs containing NP either from MDV (6 : 2 LAIV) or from wild-type virus (5 : 3 LAIV).

In vitro studies showed that both the 5 : 3 and the 6 : 2 variants of LAIV H1N1 demonstrated ts and ca phenotypes in chicken embryos; however, the 6 : 2 candidate replicated better at optimal temperature in eggs and in MDCK cells. The comparatively low replication of the 5 : 3 reassortant may be explained by a gene constellation effect; this effect is strain- or subtype-specific, since 5 : 3 LAIV reassortants of other subtypes tested by our group had similar growth profiles as the corresponding 6 : 2 reassortants [12].

Challenge studies with pandemic H1N1 cannot ethically be conducted in humans. However, mouse and ferret models are generally able to predict the pandemic potential of influenza viruses and are helpful in developing improved methods for preventing and controlling such viruses [34–36]. The current study demonstrated that new reverse genetic vaccine candidates have some residual replication capacity to replicate in mouse lungs and are indistinguishable from the parental MDV with regard to replication kinetics in the upper and lower respiratory tract of mice. Our previous results indicated that H1N1 and H7N3 LAIV 6 : 2 reassortants have some residual replication capacity in mouse lungs, unlike in the ferret model where these reassortants represent an attenuated phenotype and were not found in the lungs [28]. Safety studies in ferrets would therefore be desirable to demonstrate an attenuation phenotype.

LAIVs are capable of inducing both local IgA and serum IgG antibody responses in adult volunteers [25, 26]. It is known that serum IgG antibodies are less cross-reactive than local IgA of upper airway mucosa and provide protection mostly against matched influenza A virus strains [37]. However, serum-derived IgG of the lower respiratory tract play a key role in protection of the host against viral pneumonia [38]. In this study, we focused on serum IgG antibodies.

One or two doses of the LAIV candidates stimulated significant antibody response, as measured by HI and ELISA. The differences between the two vaccine candidates in inducing antibody response were not statistically significant; substantial levels of cross-reactive antibodies to drifted NY/wt virus were induced by both candidates. Inclusion of the wild-type NP gene in the reassortant LAIV intensified production of specific CD8+ T-lymphocytes to wild-type influenza virus. There was 100% protection from death in both the 6 : 2 and the 5 : 3 vaccine groups following lethal challenge with homologous SA/wt and drifted NY/wt viruses. Nevertheless, the 5 : 3 vaccine candidate exhibited better protective efficacy in terms of infection and severe illness than the 6 : 2 variant.

The BL6 murine lines have an immunodominant CTL response to several NP epitopes [39], which means that the C57BL6 mice can be used as models for CTL epitope immunoescape studies. Our new LAIV H1N1 candidates have differences in positions 6 and 7 of the immunodominant epitope NP_366-374_ (unpublished data). These differences in TCR-contact residues might not be critical to peptide-MHC I complex formation. Moreover, both peptides have a high predicted binding affinity to H-2(d) I class molecules (data not shown). Still, both of them could be immunogenic and induce the CTL clones with different TCR recognition moiety. Thus, LAIVs with the “old” NP gene might not induce sufficient CTL cross protection. The CTL in vivo assay showed greater clearance efficiency of influenza A virus in mice immunized with LAIV with the current NP (5 : 3 genome formula) than in mice that received LAIV with the old NP gene (6 : 2 genome formula). The latter vaccine group had similar CTL activity in vivo against wild-type influenza A virus as naive mice.

In addition to the NP-specific CTL response, there could be other vaccine-induced or immunological reasons explaining the differences in protective capacity between the 5 : 3 and 6 : 2 LAIVs. Since these vaccines differed by the source of NP protein, the anti-NP antibodies might have influenced the overall protective potential of the vaccines. Earlier study indicated that high concentrations of anti-NP IgG could protect C57BL6 mice against lethal challenge [40]. More recently, studies on influenza-exposed human sera demonstrated that anti-M1 and anti-NP antibodies can efficiently activate natural killer (NK) cells thus providing another mode of protection in response to vaccination [41]. Unfortunately, the experimental set-up did not allow distinguishing of the protective role of anti-NP antibody from that of NP-specific CTLs. Nevertheless, the overall higher cross-protective efficacy of 5 : 3 LAIV compared to 6 : 2 LAIV supports our hypothesis about the essential role of renewal of NP protein in modern LAIVs.

## 4. Conclusions

It was found that both LAIVs were similar to the MDV in terms of replication in the respiratory organs of mice and showed no neuroinvasive capacity. One dose of either LAIV candidate elicited a measurable antibody response, which was boosted by a second vaccine dose. Studies of the cell-mediated immune response in mice revealed that the 5 : 3 variant provoked greater production of specific CD8+ T-lymphocytes to wild-type influenza virus than the 6 : 2 variant. An in vivo virus-specific cytotoxic T-lymphocyte assay showed that, compared with naive mice, more effective killing of wild-type virus-infected target cells was seen in mice immunized with the 5 : 3 LAIV. These data correlate with the observed 100% protection from death in mice given either the 6 : 2 or the 5 : 3 LAIV following lethal challenge with homologous A/South Africa/3626/2013 and drifted A/New York/61/2015 viruses. The 5 : 3 LAIV candidate afforded greater protection against infection and severe illness than the 6 : 2 LAIV. Our findings confirm that both candidate LAIVs are safe and immunogenic and protect against homologous influenza virus infection in mice. Inclusion in LAIV of the NP gene from wild-type influenza virus is a new approach to inducing cross-reactive cell-mediated immune responses and cross protection against pandemic influenza.

## Figures and Tables

**Table 1:** Protection of mice against challenge with H1N1 wild-type viruses following immunization with LAIV H1N1.

Vaccine	Challenge virus								
	A/South Africa/3626/2013 (H1N1)			A/New York/61/15 (H1N1)			A/PR/8 7 : 1 rg (NP-SA) (H1N1)		
	Number died/total	% maximum weight loss	Mean virus titer in lungs, log_10_EID_50_/ml	Number died/total	% maximum weight loss	Mean virus titer in lungs, log_10_EID_50_/ml	Number died/total	% maximum weight loss	Mean virus titer in lungs, log_10_EID_50_/ml
LAIV 6 : 2	0/6	18.3	5.7 ± 0.5^*∗*^	0/6	5.9	4.6 ± 0.7	0/6	9.0	3.2 ± 0.4^*∗*^
LAIV 5 : 3	0/6	2.8	5.3 ± 0.7^*∗*^	0/6	2.8	3.8 ± 0.4^*∗*^	0/6	3.3	1.8 ± 0.4^*∗*^
Control	5/6	26.9	7.2 ± 0.3	6/6	13.8	5.8 ± 0.3	2/6	3.6	5.8 ± 1.4

^*∗*^Statistically significant at *p* < 0.05.

## Abbreviations

LAIV: Live attenuated influenza vaccine
MDV: Master donor virus
EID_50_: 50% egg infectious dose
MID_50_: 50% mouse infectious dose
HAI: Hemagglutination inhibition
GMT: Geometric mean titer
CTL: Cytotoxic T-lymphocyte
MHC: Major histocompatibility complex
TCR: T-cell receptor.
